# Methods Used to Investigate the *Plasmodium falciparum* Digestive Vacuole

**DOI:** 10.3389/fcimb.2021.829823

**Published:** 2022-01-13

**Authors:** Rebecca C. S. Edgar, Natalie A. Counihan, Sheena McGowan, Tania F. de Koning-Ward

**Affiliations:** ^1^ School of Medicine, Deakin University, Geelong, VIC, Australia; ^2^ The Institute for Mental and Physical Health and Clinical Translation, Deakin University, Geelong, VIC, Australia; ^3^ Biomedicine Discovery Institute and Department of Microbiology, Monash University, Clayton, VIC, Australia; ^4^ Centre to Impact AMR, Monash University, Monash University, Clayton, VIC, Australia

**Keywords:** *Plasmodium falciparum*, digestive vacuole, hemoglobin digestion, antimalarial, microscopy

## Abstract

*Plasmodium falciparum* malaria remains a global health problem as parasites continue to develop resistance to all antimalarials in use. Infection causes clinical symptoms during the intra-erythrocytic stage of the lifecycle where the parasite infects and replicates within red blood cells (RBC). During this stage, *P. falciparum* digests the main constituent of the RBC, hemoglobin, in a specialized acidic compartment termed the digestive vacuole (DV), a process essential for survival. Many therapeutics in use target one or multiple aspects of the DV, with chloroquine and its derivatives, as well as artemisinin, having mechanisms of action within this organelle. In order to better understand how current therapeutics and those under development target DV processes, techniques used to investigate the DV are paramount. This review outlines the involvement of the DV in therapeutics currently in use and focuses on the range of techniques that are currently utilized to study this organelle including microscopy, biochemical analysis, genetic approaches and metabolomic studies. Importantly, continued development and application of these techniques will aid in our understanding of the DV and in the development of new therapeutics or therapeutic partners for the future.

## Introduction

*Plasmodium falciparum* continues to be the deadliest form of malaria, with the World Health Organization attributing over 400,000 deaths to its infection in 2019 alone (WHO, 2020). Sadly, over 60% of these deaths are children under the age of 5, with the majority of all deaths isolated to sub-Saharan regions of Africa. Treatment of *P. falciparum* is becoming problematic as more antimalarials are losing efficacy due to the spread of drug resistant parasites. Artemisinin-combination therapies are currently considered the gold standard for *P. falciparum* treatment, but these are under threat as parasites exhibiting delayed clearance to artemisinin and resistance to partner drugs have been identified within South-East Asia, Papua New Guinea and more recently Africa, with potentially devasting effects (Hamilton et al., 2019; Miotto et al., 2020; Balikagala et al., 2021). Based on historical evidence, parasites will eventually become resistant to all antimalarials in use. Although there is a vaccine recently approved for use against *P. falciparum*, it requires multiple doses and shows less than 37% efficacy [reviewed in (Laurens, 2020)] and so there will long be a reliance for anti-malaria drugs to treat infect individuals. All of these factors continue to drive research in developing new therapeutics to tackle this disease.

**Figure 1:**
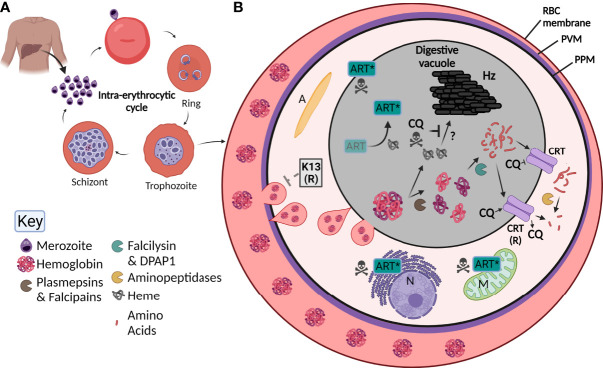
Overview of *Plasmodium falciparum* lifecycle, the digestive vacuole (DV) and the effect of antimalarials. **(A)** Upon injection into the human host, *P. falciparum* parasites initially infect and then go through a developmental phase in the liver. Parasites are then released into the blood as merozoites where they undergo the intra-erythrocytic cycle. Here, they invade red blood cells (RBC), then develop into ring stage parasites, followed by trophozoite and finally schizont stages where new merozoites are formed. Upon rupturing of the schizont, merozoites are released to propagate the cycle. **(B)** By the trophozoite stage, the digestive vacuole is visible. RBC hemoglobin (Florens et al., 2002) is taken up by the parasite through a cytostome (pink tear-shaped) and delivered to the digestive vacuole (grey) where it undergoes digestion by a number of enzymes (see key; pac-man). Small peptides are transported through the chloroquine resistant transporter (CRT) and are further digested into free amino acids. Heme (grey globin chains) is toxic and is crystallized into hemozoin (Hz) through unknown mechanisms. Chloroquine (CQ) acts by blocking the formation of Hz causing parasite death but can be overcome by mutant forms of CRT (CRT (R)) that are able to transport CQ out of the digestive vacuole. Artemisinin (Art) is activated by heme (Art*) which causes free radical damage to many organelles including the nucleus (N) and mitochondria (M). Resistance to Art is mediated by Kelch-13 (K-13), the latter involved in Hb endocytosis and trafficking to the DV. A, apicoplast; PVM, parasitophorous vacuole membrane; PPM, parasite plasma membrane.

*P. falciparum* has a complex lifecycle consisting of both a mosquito vector and its human host. An infected *Anopheles* mosquito is required to take a blood meal from a human, whereupon it injects sporozoites into the dermis (Cox, 2010). These sporozoites then travel through the blood to the liver, where they infect hepatocytes without causing any symptoms of disease. Once in the hepatocyte, exoerythrocytic schizogony occurs, where thousands of single-nucleated merozoites form. Upon rupture, these merozoites go on to individually infect red blood cells (RBC) (**Figure 1A**), often causing a range of clinical symptoms that present as flu-like with fevers and aches and can lead to anemia or more severe disease such as cerebral malaria or death. Upon invasion of the RBC, the merozoite encases itself in a parasitophorous vacuole (PV) and develops into a ring-stage parasite (Eksi and Williamson, 2011). This stage is slow growing but dynamic (Grüring et al., 2011), with parasites remodeling their host RBC by exporting hundreds of proteins into the host cytosol before transitioning into the trophozoite stage approximately 20-24 hours after invasion [reviewed in (de Koning-Ward et al., 2016)]. The trophozoite stage coincides with rapid growth and digestion of the main constituent of the RBC, hemoglobin (Florens et al., 2002), visualized by the presence of hemozoin (Hz) crystals within a digestive vacuole (DV) that is visible by light microscopy. Towards the end of the ~ 48-hour blood-stage lifecycle, daughter merozoites form and upon bursting of the mature schizont, are able to infect further RBCs, resulting in parasite amplification in the blood. Importantly, a subset of parasites within the erythrocytic cycle develop into either male or female gametocytes, where upon a further blood meal by an *Anopheles* mosquito propagates the lifecycle (Brancucci et al., 2018).

Due to the clinical manifestations of disease presenting in the RBC stages of *Plasmodium* infections, most antimalarials target this stage of the parasite lifecycle. Long before the identification of malaria, quinine, in the form of cinchona bark, was used to combat associated fevers (Achan et al., 2011), with the introduction of the closely related chloroquine in the 1940s instrumental in modern *P. falciparum* treatments. Only after the rapid onset and widespread resistance to this drug in the 1970s was it conceded that novel antimalarials were urgently required (Spencer, 1985). Analogues of both quinine and chloroquine currently used in combination therapies include piperaquine, mefloquine and lumefantrine, all of which have been reported to show resistance in clinical settings (Na-Bangchang et al., 2013; Amaratunga et al., 2016; Ljolje et al., 2018; Ross et al., 2018; Hamilton et al., 2019). The discovery of artemisinin in the 1970s provided hope to combating widespread antimalarial resistance, but its introduction has led to the appearance of parasites that exhibit delayed clearance to the drug appearing in the field (Dondorp et al., 2009; Ashley et al., 2014). Whilst artemisinin is now used only in combination therapies, parasites are developing resistance to its most common partner drug piperaquine, a chloroquine derivative (Amaratunga et al., 2016). Interestingly, the antimalarials mentioned here all appear to target or affect the function of *P. falciparum’s* DV, the site of hemoglobin digestion. Herein, this review discusses the role of the DV in antimalarial drugs with a particular focus on techniques currently used to look at the effects of both new and known compounds on the genesis and biochemical aspects of the DV.

## Digestive Vacuole Biogenesis and Constituents

The DV of *P. falciparum* was initially sighted in the 1960s using the relatively new technique for the time, electron microscopy (EM), where the authors suggested that the organelle likely contributed to Hb digestion (Rudzinska et al., 1965). This specialized acidic vacuole has since been thoroughly characterized in performing this role (Goldberg et al., 1990), although there was some conjecture as to its formation. It was originally thought that due to the DV only being visible by light microscopy from the trophozoite stage onwards, that the DV did not form until this more rapidly growing and advanced stage of the lifecycle. However, identification of Hb-containing vesicles in the mid-ring stage of development suggested that Hb was at least being taken up from the RBC cytoplasm at this stage (Dluzewski et al., 2008). It was then proposed that this begins either as a ‘big gulp’ of cytoplasm that then becomes the precursor of the DV where smaller vesicles containing Hb later traffic to (Elliott et al., 2008), or that it occurs in a completely vesicle-independent manner that relies upon actin (Lazarus et al., 2008). For these studies, both Elliott et al. (2008) and Lazarus et al. (2008) used conventional EM where no 3D configurations were used, and sample size numbers were limited. Bakar et al. (2010) refuted these claims and provided a thorough insight into DV genesis, proposing that from the mid-ring stage vesicles containing Hb are taken up and begin to act as individual DVs due to changes in their acidity and the presence of a resident protein expected in the DV. It was also demonstrated that late ring stage parasites have small but visible DVs that eventually come together to form the DV typically visible in trophozoites (Bakar et al., 2010). This version of DV genesis is now widely accepted in the field.

Once internalized, Hb is digested in the DV by proteases in an ordered process. Initially, plasmepsins (aspartic proteases) and falcipains (cysteine proteases) cleave large globin proteins into smaller polypeptides, making them more accessible for further digestion (Wunderlich et al., 2012). There are four plasmepsins located in the DV, all of which have enzymatic redundancy and can be compensated for by the falcipains (Omara-Opyene et al., 2004; Liu et al., 2006). The polypeptides produced from initial cleavage are further broken down by the two essential enzymes falcilysin and dipeptidyl aminopeptidase 1 [DPAP1; (Eggleson et al., 1999; Klemba et al., 2004b)]. Finally, free amino acids appear to be cleaved from small or dipeptide chains by four aminopeptidases. It has recently emerged that this final step of Hb digestion occurs outside of the DV itself (Mathew et al., 2021) (**Figure 1B**). Overall, Hb digestion plays important roles in the parasite’s free amino acid pool, in regulating osmotic pressure, and providing physical space for the growing parasite (Lew et al., 2004; Liu et al., 2006). Throughout this whole process, large quantities of the toxic metabolite heme are produced, that without detoxification causes cellular damage and death (Campanale et al., 2003). To overcome this, parasites transform heme into an inert hemozoin (Hz) crystal through mechanisms that are still poorly understood but may involve enzymes involved in Hb digestion itself (Chugh et al., 2013; de Villiers and Egan, 2021) (**Figure 1B**). Hz within the DV is visible under light microscopy as a dark punctate structure within parasites and upon schizont rupture it is released from the parasite(s) as waste (Dasari et al., 2012).

## The Digestive Vacuole as a Drug Target

It is now apparent that most antimalarials in use target some biological aspect of or within the DV. Chloroquine has been thoroughly characterized as a Hz inhibitor, where treatment results in DV swelling and eventual parasite death (Wicht et al., 2020). Initial characterization of chloroquine’s involvement with the DV was established in the late 1990s, when intact DVs were isolated and purified from parasites before being incubated with radioactive chloroquine and the drug could be detected in these vacuoles (Saliba et al., 1998). It is now known that the drug passively accumulates in the DV due to its acidic nature, and forms a complex with Hz on the growing crystal face, preventing further detoxification of accumulating free heme that causes catastrophic damage to the cell (Olafson et al., 2015). Treatment with chloroquine has been shown to increase the amount of free heme within parasitized RBCs as a whole, as well as inhibiting the formation of synthetic Hz in cell-free systems, supporting this MOA which is now well established (Ncokazi and Egan, 2005; Combrinck et al., 2015). Characterization of chloroquine accumulation in isolated DVs from both sensitive and resistant parasite lines showed that DVs from resistant parasites accumulated less chloroquine, the first indication that the DV was involved in the drug’s resistance (Saliba et al., 1998). A transporter located in the DV membrane, the *P. falciparum* chloroquine-resistance transporter (*Pf*CRT), is responsible for transporting short peptide chains originating from Hb into the parasite cytoplasm (Shafik et al., 2020). This transporter has long been associated with chloroquine resistance, with mutations observed in the field responsible for actively transporting chloroquine out of the DV, preventing it from interacting with Hz and thus avoiding its MOA (Fidock et al., 2000; Martin et al., 2009). A wide range of mutations in *Pf*CRT have been identified that ultimately resulted in widespread resistance to this therapeutic (Callaghan et al., 2015; Kim et al., 2019).

Dihydroartemisinin (DHA), the active metabolite found in all artemisinin derivatives, has a somewhat more convoluted MOA. It has been shown that treatment with DHA has a multi-target effect on the processes of glycolysis, cell cycle regulation, protein turnover and Hb uptake (Wang et al., 2015; Ismail et al., 2016; Bridgford et al., 2018). DHA passively accumulates in the DV and is activated by heme, displaying killing action against both trophozoite and early ring stage parasites, suggesting that at this early stage Hb is being digested in order for heme to activate the drug [reviewed in (Tilley et al., 2016)]. Some findings also suggests that the drug could initially be activated by heme synthesized by the parasite in the cytosol within the early stages of development rather than heme solely originating from Hb digestion (Wang et al., 2015). Once activated, DHA forms a radical complex that quickly causes damage to parasite proteins, whilst also causing a cascade of further free radicals to develop, eventually leading to parasite death (Bridgford et al., 2018). Due to the nature of this MOA, it is not surprising that a number of processes are affected after treatment. A range of molecular markers conferring artemisinin resistance have been identified, with the overriding consensus being that particular mutations in Kelch-13 play an important role in delayed clearance of parasites after drug treatment (Ariey et al., 2014; Straimer et al., 2015). The natural function of Kelch-13 is in the endocytic uptake of Hb from the host cell cytoplasm, and a range of mutations present in this protein have been shown to lead to a decrease in Hb uptake (Huang et al., 2015; Birnbaum et al., 2020; Bwire et al., 2020). In the study by Birnbaum et al. (2020), other members of a non-canonical endocytic pathway in addition to Kelch-13 were also identified using a proximity ligation technique and these included epidermal growth factor receptor substrate-15 (Eps15), ubiquitin carboxyl-terminal hydrolase (UBP1), adaptor protein-2 (AP-2), and a range of Kelch-13 interaction candidates (KIC proteins). Inactivation of a number of these proteins resulted in a decrease in Hb uptake and artemisinin resistance. Since DHA is activated by Hb digestive products, decreasing uptake and thus digestion of this RBC constituent would result in decreased activation of the drug and generation of parasite resistance as well as to a perturbed parasite lifecycle (Hott et al., 2015). However, as Hb digestion itself is an essential process, this mechanism of resistance is likely to be self-limiting and may only result in delayed clearance rather than complete resistance. In the field, artemisinin resistance has in fact been reported as an increase in parasite clearance half-life rather than an increase in drug required for treatment, supporting this hypothesis of Kelch-13 induced resistance (Wicht et al., 2020). Other less understood mechanisms of artemisinin resistance have been reported, with many of these also appearing to be involved in Hb uptake or digestion (Wang et al., 2016; Rocamora et al., 2018).

All considered, it is evident that Hb digestion and the DV are an attractive and well utilized drug target of antimalarials. Due to the essential nature of the processes occurring here, and the fact that the DV is highly parasite-specific, development of novel compounds that target the DV have the potential to complement current therapeutics. It is important that the MOA of new compounds is well understood to combat potential resistance and to ensure compatibility with partner drugs. As outlined below, this has required the use of new and more advanced technologies to tease apart the structure and function of the DV and its constituents to provide further insight into which may serve as attractive targets for future therapeutic development.

## Microscopy Approaches to Analyze DV Morphology

In more recent times microscopy has been a fast moving and evolving field, and for a review on microscopic techniques used to study *Plasmodium* species as a whole we refer the reader to (De Niz et al., 2017). With respect to the DV, microscopy has provided a powerful tool to analyze its structure and function under different treatments, with techniques discussed here summarized in **Table 1**. Standard microscopy techniques are still routinely used, with Giemsa-staining and basic light microscopy able to characterize the size and volume of DVs. For example, a range of compounds, including chloroquine, induce the swelling of DVs as water osmotically enters after the deregulation of Hz formation and this is evident by light microscopy (Prasad et al., 2013; Pulcini et al., 2015). Whilst crude parasite images of such phenotypes are informative, without further investigation they alone provide little insight into the biochemical process or how the drugs disrupt DV morphology. Using higher resolution differential interference contrast (DIC) microscopy allows for clearer analysis of DV development (Bakar et al., 2010) and aberrant phenotypes such as DV bloating can be visualized (Sijwali and Rosenthal, 2004; Jonscher et al., 2019), especially when paired with fluorescent probes and computational reconfiguration. This provides helpful insights into natural genesis and potential drug effects on the DV. Of even higher resolution is confocal microscopy, which is able to reduce background or non-focused light away from the field of interest, providing the ability to take stacked images to provide 3D and even 4D images of *P. falciparum* and the DV (Grüring et al., 2011).

**Table 1:** Summary of microscopy techniques used to image digestive vacuoles and its constituents in *Plasmodium falciparum*.

Technique	Advantages	Disadvantages	Examples
Light Microscopy	Cheap equipment, easy to use	Relatively low resolution	(Prasad et al., 2013; Pulcini et al., 2015)
Differential Interference Contrast (DIC) Microscopy	Live and fixed imaging available	3D images may not be accurate	(Bakar et al., 2010; Jonscher et al., 2019)
Confocal Microscopy	Reduced background light for higher resolution than DIC, fluorescence imaging	Affected by some cells natural fluorescence e.g. Hemozoin	(Grüring et al., 2011; Lee et al., 2014)
Spinning Disk Confocal Microscopy	Ability to image thick samples for high resolution 3D images, faster imaging speed	Limited by available fluorescent lasers	(Gligorijevic et al., 2006a; Gligorijevic et al., 2006b)
Stimulated emission depletion microscopy (STED)	Live and fixed fluorescence imaging available, embedding and slicing not required	Expensive equipment and training required	(Schloetel et al., 2019)
Electron Microscopy (EM)	Nanometer resolution	2D resolution, embedding and slicing required, expensive and specialized equipment and training required	(Ch’Ng et al., 2011; Asad et al., 2021)
Electron Tomography	Nanometer resolution without fixature maintains native state of cells and organelles	Expensive and specialized equipment and training required	(Bakar et al., 2010)
X-ray tomography	Slicing not required for imaging as with EM	Expensive and specialized equipment and training required	(Hanssen et al., 2012; Kapishnikov et al., 2013)

EM is still widely used to assess DVs, with entire organelles within the parasite identifiable with the use of immune labelling to help identify the DV and areas within the DV (Asad et al., 2021). The treatment of parasites with different compounds often leads to a range of DV disruptions that can also be distinguished. Chloroquine treatment, for example, results in a range of DV abnormalities including loss of ordered Hz crystals and/or permeabilization or complete loss of the DV membrane (Ch’Ng et al., 2011). Treatment with an apicoplast inhibitor, indolmycin, revealed fragmentation of the DV, yet they still contained Hz and appeared to be functional as individual DVs. This could be confirmed with scanning EM (SEM) coupled with 3D computational rendering, showing that apicoplast disruption does in fact lead to multiple DVs developing per parasite (Kennedy et al., 2019). Although EM provides detailed and high-resolution images, they are in 2D unless 3D reconstructions are assembled after images are taken. Electron tomography, on the other hand, gives the ability to see 3D preserved structures within cells without the harshness of fixatives, and thus is more likely to provide images of parasites and organelles closer to their natural state (Lučić et al., 2008; Bakar et al., 2010). *P. falciparum* parasites imaged this way have revealed that Hb uptake is initiated in the early stages of parasite development (Bakar et al., 2010). Similarly, X-ray tomography is also able to eliminate the need for harsh fixatives and has the additional benefit of being able to image thicker samples than what is possible with electron microscopy. While samples still need cryo-preservation as is the case for electron tomography, they do not need slicing, and samples near native state are able to be imaged and seen as 3D structures. Both Hb (Hanssen et al., 2012) and Hz (Kapishnikov et al., 2012) have been visualized in infected RBCs using this method, as well as regions of the DV itself (Kapishnikov et al., 2013). The localization and effect of the antimalarial quinine has also been analyzed using X-ray tomography, with authors showing the compound binds to forming Hz crystals in a way that prevents further heme crystallization (Kapishnikov et al., 2019). These advanced microscopy techniques have provided very detailed and important information about the DV, especially in the scheme of looking at effects of new and known antimalarial compounds. Nevertheless, there are limitations to these approaches, including the extensive training time and cost required for technicians, as well as the cost of running and maintaining instruments and the fact that they provide no information on the biochemical processes occurring within the DV itself.

The addition of fluorescence labelling technologies in conjunction with the wide range of microscopy techniques available allows analysis of DV structure with the added bonus of being able to assess potential function. At the very basic level, immunofluorescence analysis of proteins tagged with epitope tags or reporter genes in fixed or live cells, can provide information on the localisation of known and novel parasite proteins, particularly in relation to the proximity of the DV when used in conjunction with DIC or known DV markers (Klemba et al., 2004a; Bakar et al., 2010; Ehlgen et al., 2012; Agrawal et al., 2020). DV morphology and/or native protein localisation after drug treatment can also be determined when fluorescently tagged proteins that typically reside in the DV are affected (Gisselberg et al., 2017). However, these require either some knowledge of the target that is being probed for or time and skill to generate transgenic parasites, but in comparison to other techniques may provide a cheaper alternative. Confocal microscopy is typically employed when visualizing fluorescent or epitope-tagged *P. falciparum* proteins, providing relatively clear images of both live and fixed samples. Stimulated emission depletion microscopy (STED) improves on confocal resolution but has been known to cause significant damage to samples, particularly as Hz is reactive to many fluorescent lasers. In order to overcome this, Schloetel et al. (2019) developed a guided STED approach that reduced damage to cells and Hz, allowing for super high resolution fluorescent images where Hz itself would also be imaged due to its natural fluorescence (Schloetel et al., 2019).

When looking at the effect of compounds on either Hb uptake or the DV itself, it can be particularly useful to fluorescently stain the RBC cytoplasm or the Ca^2+^ content of the DV. A wide range of labeled Dextran probes are commercially available, which offer the ability to fluorescently label the RBC cytoplasm prior to infection with *P. falciparum*. Once infected, any cytoplasm that is taken up by the parasite can be identified under fluorescent microscopy, and such approaches have provided insight into abnormal formation of the DV after treatment with the apicoplast inhibitor, indolmycin (Kennedy et al., 2019) or with phenotypic analysis of genetically modified parasites to elucidate protein function (Jonscher et al., 2019). Gligorijevic et al. (2006a) utilized dextran-conjugated oregon green to fluorescently label the DV of parasites resistant and sensitive to the antimalarial chloroquine, finding that resistant parasites developed relatively larger and more acidic DVs (Gligorijevic et al., 2006a). At the time, they used the novel technique of spinning disk confocal microscopy (SDCM) to image malaria parasites, which provided 3D images over time, thus negating problems often present when using conventional confocal microscopy due to the constant movement of parasites within their RBC. This allowed visualization of Hz formation (Gligorijevic et al., 2006b) and DV morphology (Gligorijevic et al., 2006a) under physiological conditions, with the technique further utilized to study the effect of some drug resistant markers that may impact the DV (Lee et al., 2018). Another method of fluorescently labeling the DV that has become more popular is the use of Fluo-4-AM, a probe that intensifies in emission 100-fold upon binding to calcium, of which the DV is the main reservoir of in *P. falciparum* (Biagini et al., 2003). Of interest, this probe has been used in a range of drug screening scenarios. Parasites treated with compounds that result in a disrupted DV lead to the release of calcium-bound Fluo-4-AM into the parasite cytoplasm, which in turn causes a shift in fluorescence that can be detected by flow cytometry and confirmed by fluorescence microscopy; screens looking at known or novel antimalarials not only thought to target the DV but also Ca^2+^ dynamics have been performed using Fluo-4-AM (Lee et al., 2014; Chia et al., 2017; Tong et al., 2018; Chia et al., 2021). This probe is also helpful for more specific phenotypic and localisation studies, and confocal microscopy has been used to access the effect of antimalarials on the DV in parasites harboring chloroquine or multi-drug resistance (Ch’Ng et al., 2011).

Fluorescent probes that are pH dependent are also able to utilize the acidic nature of the DV to fluorescently label them, with probes such as Lysotracker or LysoSensor commonly used in microscopy (Lin et al., 2001; Bakar et al., 2010; Bohórquez et al., 2012; Agrawal et al., 2020). Bakar et al. (2010) utilized SNARF-1-dextran, a pH-sensitive fluorescent probe, on infected RBCs to thoroughly characterize the formation of vesicles and the DV through the ring and trophozoite stage of *P. falciparum*. As the RBC cytoplasm became internalized and acidified, a color shift was identifiable under confocal microscopy, which was also confirmed by Lysosensor-Blue and green fluorescent protein (GFP) tagging of a known DV protein (Bakar et al., 2010). This study provided important information on the biogenesis of the DV in the earlier stages of parasite development. Changes in the pH of the DV after treatment with compounds can also be investigated using these probes (Saha et al., 2019), as well as overall parasite pH changes after treatment with known antimalarials (Tang et al., 2019).

## Biochemical Analysis of Hb and Its Digestive Products

In conjunction to examining the DV using a range of microscopic techniques it is important to also analyze the process of Hb digestion and its byproducts when characterizing antimalarials or potential drug targets. Crude characterization can be performed on whole parasite lysates to determine if compounds or drugs affect Hb digestion. In this case, lysates are electrophoresed on SDS-PAGE gels and stained for protein, with Hb accumulating as a low molecular weight band that can be imaged and quantitated relative to lysates from parasites treated with vehicle and/or positive controls (Prasad et al., 2013).

The ability of Hb and heme to be measured spectroscopically also provides an easy and accessible way to measure the different species found within parasites. An advanced technique developed by Combrinick et al. (2013) allowed for the analysis of the percentage of Hb, Hz and free heme within parasites after treatment with chloroquine, demonstrating that Hz formation is impaired and free heme increases upon treatment, as is established as the drugs MOA. This workflow has been further optimized to develop a high throughput colorimetric screen that has been validated with chloroquine (Combrinck et al., 2013) and applied to a number of other known antimalarials, providing novel insight into their effect on Hb, Hz and free heme species (Combrinck et al., 2015). This screen has been applied to parasites harboring known drug resistant markers (Gabryszewski et al., 2016) and can provide important information when analyzing novel drugs (Birrell et al., 2020). While this assay has been developed into a high throughput workflow, it is still somewhat complex. Men et al. (2012) showed that purification of Hz, followed by its conversion into free heme which was then measured *via* colorimetric analysis, was linearly related to parasitemia (Men et al., 2012). Although this assay provides less information than the method described above, it is a simpler and more cost-effective way of examining the amount of Hz under different drug treatments, indicating if Hz production and thus Hb digestion has been affected (Subramanian et al., 2019; Edgar et al., 2021). Quantification of the amount of Hz in parasites can also be performed using a standard curve of β-hematin, a validated synthetic form of Hz (Tripathi et al., 2004). Asad et al. (2021) used commercial β-hematin to create a standard curve to elucidate the effects of Hz production following knockout of a number of parasite genes and drug treatment on parasites, providing insight into their overall effect on the Hb digestion pathway (Asad et al., 2021).

Recombinant expression of functional parasite proteins of interest can also provide indirect information as to their relationship with Hb digestion and the DV. When incubated with Hb, recombinant falcipain-2 (a now characterized DV enzyme) was shown to hydrolyze Hb in a cell-independent manner (Pandey et al., 2005). The interactions that novel compounds have on Hb and its substituents are also able to be performed in a cell-independent manner. High throughput screening of β-hematin formation allows for the identification of compounds that inhibit this, which may be helpful in identifying compounds that may have antimalarial activity (Ncokazi and Egan, 2005). This assay has now been widely used. Saha et al. (2019) used both synthetic heme and human Hb to investigate the actions and interactions of their novel antimalarial compound, before confirming findings on parasites and lysates (Saha et al., 2019). This enabled the determination of a potential MOA even before the compound was used on parasites, although the latter is still important to confirm the cell-free results. Birrell et al. (2020) used β-hematin formation inhibition to further show that their novel antimalarial compound effected Hb digestion independent of Hz formation. The ability of compounds to inhibit the formation of Hz, particularly when used alongside a positive control such as chloroquine (Subramanian et al., 2019), is now a helpful tool in determining the effect of novel compounds on this essential process and to identify compounds with new modes of action that still target Hb digestion and the DV.

## Genetic Approaches

Understanding the biological role and essentiality of DV proteins is paramount in determining their suitability as therapeutic targets. Genetic knockout provides direct information on this, and indeed the proteases involved in the earliest stage of Hb digestion, the plasmepsins, were found to be functionally redundant using this approach (Bonilla et al., 2007). Analysis of plasmepsin knockout parasites performed by Bonilla et al. (2007) determined that a triple knockout, where three plasmepsins were knocked out in one parasite line, had no effect on parasite growth and survival compared to wildtype parasites, but that a quadruple knockout resulted in a severe growth defect. Falcipain-2 was also amenable to genetic knockout on both a wild-type and plasmepsin double knockout background (Liu et al., 2006). The knowledge that these enzymes are not essential for Hb digestion and survival removed them as suitable drug targets. Attempts have also been made to knockout several genes that encode enzymes involved in later stages of Hb digestion, including DPAP1 and aminopeptidases, most of which appear to be essential for survival (Klemba et al., 2004b; Dalal and Klemba, 2007). Large scale screening for essential proteins has also been performed on *P. falciparum* using saturation mutagenesis with piggyBac transposons (Zhang et al., 2018). Whilst a limitation of this strategy is that smaller genes may be annotated as essential due to the frequency of transposon insertions in the genome, it provides a good starting point when accessing if a protein is suitable as a therapeutic target.

In order to functionally assess proteins that are essential to the blood stages, conditional regulatory systems are required. For reviews on genetic approaches used throughout *Plasmodium* research we direct readers to (de Koning-Ward et al., 2015; Kudyba et al., 2021; Okombo et al., 2021). A number of these systems now exist and include regulation at the DNA, transcript, and protein level. Knockdown of falcipain-3 using the *glmS* ribozyme system, which works by disruption of mRNA after the addition of an inducer, determined that loss of the protein had no effect on parasite growth (Xie et al., 2016). The aminopeptidase M17, one of four enzymes implicated in the end stage of Hb digestion, was determined to be essential for parasite survival using the ribozyme system. Analysis of parasites depleted of the protein showed that parasite death was likely due to a buildup of undigested peptides originating from Hb (Edgar et al., 2021). However, knockdown using this method rarely results in complete loss of the protein and may result in no detectable phenotype even when the expression of an essential gene is knocked down. Thus, conditional knockout systems may provide an alternate approach to assess function. AP-2, which is involved in Hb uptake and trafficking to the DV as well as artemisinin resistance (Birnbaum et al., 2020), has been characterized using the conditional di-Cre knockout method, which demonstrated this protein is essential and is involved in clathrin-independent trafficking (Henrici et al., 2020). Many other interacting partners or enzymes involved in Hb digestion have yet to be studied using these conditional methods, and instead studies have utilized small molecule inhibitors to gain insight into protein essentiality and function. While useful, they still pose the risk of having off-target effects (Deu et al., 2010; Harbut et al., 2011) and thus results should be validated using genetic approaches.

## Recombinant Expression Systems

Parasite cell free systems have provided a popular method to examine Hb and its digestion products and can provide further information into the make-up of the DV itself. The *Xenopus* oocyte system has been well utilized to determine the function of *Pf*CRT – its expression in this heterologous system confirmed that mutated *Pf*CRT can efflux chloroquine while the wild-type transporter cannot (Martin et al., 2009). This system has been further used to examine particular mutations that have been found in field isolates to determine their contribution to the function of efflux and consequently the effect this would have on their ability to transport chloroquine (Pulcini et al., 2015). Furthermore, this system has been used to elucidate that the natural function of *Pf*CRT is a peptide transporter, as well as playing an important role in iron homeostasis (Bakouh et al., 2017; Shafik et al., 2020). However, expression of genes in the *Xenopus* oocyte system is highly variable and requires significant investment in skills and facilities. Nonetheless, it can provide important information as to whether novel compounds that target the DV are also likely to be effluxed by this mechanism. Other transporters that are also likely to be involved in drug resistance can be analysed in this way before or in conjunction with analysis performed on parasites themselves.

The recombinant expression of parasite proteins in *Escherichia coli* or other heterologous expression systems also provides an indirect way to study constituents of the DV. In addition to the characterization of recombinantly expressed falcipain-2 as discussed above, recombinant plasmepsin histo-aspartic proteinase (HAP) has been shown to hydrolyze globin peptides (Xiao et al., 2006). The enzymatic specificity of two aminopeptidases theorized to be involved in end-stage Hb digestion has also been assessed using recombinantly expressed proteins whilst also utilizing fluorescence to demonstrate enzymatic activity (Poreba et al., 2012; Malcolm et al., 2021). Further to functional analysis of recombinantly expressed proteins and their enzymatic mechanism(s), structural biology techniques can provide important atomic information on drug binding pockets as well as map the binding of novel compounds within target proteins to aid in compound design and optimization (Prade et al., 2005; Bhaumik et al., 2009; Mistry et al., 2014).

## Metabolomics

Whole cell metabolomics have also become an attractive platform to analyze the effects of known or novel compounds on Hb digestion, and for a review on metabolomics used in all *Plasmodium* research we refer readers to (Yu et al., 2021). Using nuclear magnetic resonance or mass spectrometry to analyze metabolites present in *P. falciparum* parasites after drug treatments presents an indirect way to examine their effects on processes within the parasite, including Hb digestion. A build-up or loss of peptides found in Hb can represent downstream or upstream blocking of this digestion pathway, respectively and to date has provided useful information into the effect of novel or potential repurposed compounds on Hb digestion (Birrell et al., 2020; Burns et al., 2020; Giannangelo et al., 2020; Edgar et al., 2021). Metabolomic analysis after DHA treatment has been shown to result in a decrease in Hb-derived peptides, in accordance with resistance to this drug being attributed to a decrease in Hb uptake (Mok et al., 2021). Multiple screens have also been performed on compound libraries where metabolites have been identified as probable Hb-derived peptides, enabling authors to conclude that their accumulation after drug treatment is representative of the compound targeting or having an effect on Hb digestion (Allman et al., 2016; Creek et al., 2016). Characterization of compounds with known modes of action complement this work and confirms metabolomics as a useful tool in drug characterization (Cobbold et al., 2015). Metabolomics has also been used to investigate profiles of chloroquine-resistant parasites in terms of what effects mutations in *Pf*CRT potentially have on Hb digestion compared to wildtype transporters. Parasite lines either episomally expressing resistant forms of *Pf*CRT or lines that harbor resistant alleles have all been shown to accumulate a higher number of short, hemoglobin-derived peptides, suggesting that mutations have a fitness cost on the natural function of *Pf*CRT (Lewis et al., 2014; Gabryszewski et al., 2016; Lee et al., 2018). Being able to determine Hb-derived peptide accumulation or loss after compound treatment is a valuable way to determine their involvement in Hb digestion, however, it is important to note that it cannot distinguish if this is a result of directly targeting Hb digestion or a result of parasite death through potentially unrelated mechanisms.

## Concluding Remarks

The development of *P. falciparum* resistance to antimalarial drugs continues to drive research into discovering and identifying the MOA of new and novel compounds. The parasite’s DV is a well utilized target of many currently used therapeutics and should continue to be one, given that unique and essential processes occur in this organelle. The identification of compounds with novel modes of actions within the Hb digestion pathway provide an opportunity to complement current therapies that may be under resistance pressure. A complete understanding of such compounds is bound within the constraints of experimental systems and research should always be attempting to move with the improvements within and external to established protocols. Rapidly improving microscopy techniques are being well utilized to determine structural and thus potentially functional responses to compound treatment as well as being applied in high throughput screening approaches. Biochemical analysis is also being incorporated in higher throughput protocols, and as compound identification continues to be pushed towards drug repurposing, such protocols should continue to be developed and utilized to help understand their potential mechanisms of action on *Plasmodium* parasites. Genetic approaches to ascertain protein essentiality and function has been able to discern which processes in the DV are essential or redundant for parasite survival, enabling further identification of possible new antimalaria targets.

## Author Contributions

RE, NC, SM, and TdK-W were responsible for writing and editing the manuscript and approved the final version.

## Conflict of Interest

The authors declare that the research was conducted in the absence of any commercial or financial relationships that could be construed as a potential conflict of interest.

## Publisher’s Note

All claims expressed in this article are solely those of the authors and do not necessarily represent those of their affiliated organizations, or those of the publisher, the editors and the reviewers. Any product that may be evaluated in this article, or claim that may be made by its manufacturer, is not guaranteed or endorsed by the publisher.
